# Artificial intelligence for clinical decision support for monitoring patients in cardiovascular ICUs: A systematic review

**DOI:** 10.3389/fmed.2023.1109411

**Published:** 2023-03-31

**Authors:** Sobhan Moazemi, Sahar Vahdati, Jason Li, Sebastian Kalkhoff, Luis J. V. Castano, Bastian Dewitz, Roman Bibo, Parisa Sabouniaghdam, Mohammad S. Tootooni, Ralph A. Bundschuh, Artur Lichtenberg, Hug Aubin, Falko Schmid

**Affiliations:** ^1^Digital Health Lab Düsseldorf, Department of Cardiovascular Surgery, Medical Faculty and University Hospital Düsseldorf, Düsseldorf, Germany; ^2^Institute for Applied Informatics (InfAI), Dresden, Germany; ^3^Department of Computer Science, Heinrich-Hertz-Europakolleg, Bonn, Germany; ^4^Department of Health Informatics and Data Science, Loyola University Chicago, Chicago, IL, United States; ^5^Nuclear Medicine, Medical Faculty, University Augsburg, Augsburg, Germany

**Keywords:** artificial intelligence (AI), machine learning (ML), clinical decision support (CDS), cardiovascular, intensive care unit (ICU), patient monitoring, explainable AI (XAI)

## Abstract

**Background:**

Artificial intelligence (AI) and machine learning (ML) models continue to evolve the clinical decision support systems (CDSS). However, challenges arise when it comes to the integration of AI/ML into clinical scenarios. In this systematic review, we followed the Preferred Reporting Items for Systematic reviews and Meta-Analyses (PRISMA), the population, intervention, comparator, outcome, and study design (PICOS), and the medical AI life cycle guidelines to investigate studies and tools which address AI/ML-based approaches towards clinical decision support (CDS) for monitoring cardiovascular patients in intensive care units (ICUs). We further discuss recent advances, pitfalls, and future perspectives towards effective integration of AI into routine practices as were identified and elaborated over an extensive selection process for state-of-the-art manuscripts.

**Methods:**

Studies with available English full text from PubMed and Google Scholar in the period from January 2018 to August 2022 were considered. The manuscripts were fetched through a combination of the search keywords including AI, ML, reinforcement learning (RL), deep learning, clinical decision support, and cardiovascular critical care and patients monitoring. The manuscripts were analyzed and filtered based on qualitative and quantitative criteria such as target population, proper study design, cross-validation, and risk of bias.

**Results:**

More than 100 queries over two medical search engines and subjective literature research were developed which identified 89 studies. After extensive assessments of the studies both technically and medically, 21 studies were selected for the final qualitative assessment.

**Discussion:**

Clinical time series and electronic health records (EHR) data were the most common input modalities, while methods such as gradient boosting, recurrent neural networks (RNNs) and RL were mostly used for the analysis. Seventy-five percent of the selected papers lacked validation against external datasets highlighting the generalizability issue. Also, interpretability of the AI decisions was identified as a central issue towards effective integration of AI in healthcare.

## 1. Introduction

Complications due to clinical deterioration and medical errors are often caused by human error, either due to forgetfulness, inattention, or inexperience and are far greater than technical failures (1, 2). Furthermore, intensive care units (ICUs) are prominent sources of large bulk of data collected from each patient. For the special case of cardiovascular ICU patients who mostly attribute higher complication rates and longer ICU stays (3, 4), it becomes even more challenging for the medical staff to spot certain complications or symptoms of patients. Considering the promising impact of artificial intelligence (AI) for clinical decision support (CDS) (5, 6), implementing AI into the cardiovascular ICUs could help minimize the number of medical errors by being able to guide the clinician to the correct diagnosis and ultimately to an appropriate therapy.

In the context of medical AI, the two major disciplines of Medicine and AI need to come together. Recent discoveries in medicine and medical technology as well as new advancement in AI modeling and computational power increased the application of ML-based methodologies in healthcare domains, such as disease diagnosis, prognosis and treatment planning (7–10), and overall/disease-free survival prediction (11–13). In particular, in intensive patient monitoring, AI methods have been used for different purposes such as prediction of readmission (3, 14–16) and sepsis (17–19) and mortality risk assessment (20, 21).

Despite the large body of evidence illustrating the promising relevance of AI methodologies in medical domains, there are some common challenges which limit the integration of AI-based methodologies in daily routines. For instance, trained classifiers may make biased predictions due to various sources of bias, such as gender bias, present in medical datasets (22, 23). Another challenge is the ‘black box’ nature of most of the modern deep and recurrent neural network models, which necessitates solutions to address explainability of these methods when applied to medical domains (24). Furthermore, ensuring consistency between the characteristics of open access data sets used for training and real clinical data is crucial for the successful integration of AI in intensive care routine practice (25). We aimed to draw attention to the limitations stemming from bias, interpretability, and data set shift issues, which expose a gap in the integration of AI in clinical decision making. This gap is mostly caused by medical staff’s lack of trust in AI.

There are already a number of impactful articles which closely relate to the current systematic review. Fleuren et al. (26) conducted a systematic review and meta-analysis of AI models to predict sepsis onset in different wards including normal, emergency and ICU stations. Although their findings illustrate that ML models can achieve high accuracy in predicting sepsis in their corresponding experimental setups and might be considered as alternatives to some established scoring systems in clinical routines, they identify a lack of systematic reporting and clinical implementation studies in the domain which should be overcome in the future. Giordano et al. (27) argued that patient risk stratification and patient outcome optimization would be the first venues in which AI can practically contribute to routine practices. However, the mentioned work emphasizes the necessity for medical staff to receive extracurricular training on the mechanics of AI decision making and improved interpretability. This can ultimately lead to increased trust in AI in healthcare scenarios. Syed et al. (28) identified that predicting mortality, sepsis, acute kidney injury (AKI), and readmissions were the most common tasks for applied AI in patient monitoring in ICUs. Greco et al. (29) identified inconsistencies in diagnosis and treatment protocols between different health centers and countries as well as the lack of emotional intelligence to be the most critical aspects which confine the successful integration of AI driven approaches for patient monitoring. Antoniadi et al. (24) addressed interpretability as one of the most critical issues towards integration of ML-based approaches for CDS, identifying tabular data processing XAI-enabled systems and XAI-enabled CDS tools for text analysis as the most and the least common approaches in the literature, respectively. Also, Yang et al. (30) addressed the medical XAI aspects in multi-modal and multi-center scenarios in a mini-review study. They further showcased an XAI framework integrated for automated classification of corona virus disease (COVID)-19 patients and ventricle segmentation using computed tomography (CT) and magnetic resonance imaging (MRI) scans. Finally, Abdellatif et al. (31) reviewed the applications of reinforcement learning (RL) for intelligent healthcare (I-Health) systems, focusing on large networks of Internet of mobile things (IoMT) and software defined networks (SDNs) producing big data. In the realm of this evolving field, our work distinguishes itself by emphasizing the strategies and knowledge necessary to bridge the gap and successfully integrate AI for clinical decision support in daily intensive care routines, with a particular focus on cardiac diseases.

In this systematic review, following the PRISMA (32) and PICOS (33) guidelines, we designed the study in four steps including: identification of initial manuscripts through search engine queries and subjective searches, screening of original articles upon availability of full text in English, eligibility with regard to domain of interest and technical significance as well as medical relevance of the studies. We considered the most well-known publisher databases in the clinical and medical research domains to search and select high quality original research articles. We mainly focused on shortlisting the works that aimed at analyzing the applications of AI-assisted methodologies for automated patient monitoring in cardiovascular ICUs. We further analyzed most common data types as well as mostly applied AI algorithms for decision support in patient monitoring. The main contributions of this manuscript can be listed as following:

- Performing a systematic review over patient monitoring articles following PRISMA and PICOS guidelines
- Covering the technical foundations according to the medical AI life cycle (34)
- Providing an extensive factual and narrative analysis of the selected articles
- Providing expertise from both data science and medical science points of view
- Discussing limitations and insights for the successful integration of AI-driven methods for decision making in cardiac ICUs
- Recommending additional standardization and risk of bias criteria applicable to novel medical AI tools with regards to generalization and external validation aspects.

In the next sections, first, we discuss the basic concepts which are fundamental to be able to follow the reported findings from the selected articles. The Methods section provides the details on the screening and selection criteria of the papers followed by the Results and Discussion sections which provide a comprehensive outline of the findings from the selected contributions. Finally, a short conclusion of the findings is given.

## 2. Background and fundamental concepts

According to the best practices (34), the life cycle of medical AI includes (a) model development and evaluation, (b) data creation and collection, and (c) AI Safety. Therefore, we covered the current state of the methods used in major related work, the data used in the studies, and the recent advances in the interpretability and explainable AI for medicine. The rest of this section briefly describes some of the most important concepts in these three aspects which are critical for better understanding of the topics that are covered in the next sections. Note that, the choice of methods which are discussed in this section reflects the methodology implemented in the selected articles as a result of the systematic review process.

### 2.1. Common AI methods applied to clinical data for patient monitoring

From a high-level perspective, machine learning (ML) techniques can be categorized in three main groups: supervised, unsupervised and reinforcement learning. If ground truth labels are available and used to train and fit the model (e.g., binary classification using known classes), the model corresponds to supervised ML paradigm. Otherwise, if the model is trained without prior knowledge on the target variable (e.g., clustering), the model corresponds to unsupervised ML paradigm. Another ML paradigm that has been frequently used for clinical decision support is Reinforcement Learning (RL).

*Reinforcement learning*: In RL, a computational agent is trained to maximize the cumulative reward it receives over a series of time-steps by taking observations of the current state of the environment and by evaluating the feedback it receives after taking an action in that state (35). More formally, RL is founded on a Markov Decision Process (MDP) (36), where the RL agent is trained to learn an optimal policy pi* that maximizes the cumulative reward by exploring the environment defined by *p(s, a, s’)* and *r*, and exploiting its knowledge of the environment represented by *V_pi* or *Q_pi* and *y*.

There is a long history of clinical decisions being formulated as an MDP. Initial efforts in this direction focused on dynamic programming solutions, while in recent work, variations of the Q-Learning algorithm have become more prominent, such as fitted-Q-iteration (FQI) (37) or deep Q-networks (DQN) (38). Areas where RL has been applied, that are relevant for cardiovascular monitoring include targeting of measurements during monitoring and choosing, timing and dosing of treatment steps. Many diagnostic and prognostic tasks in the healthcare domain are facilitated through the use of a variety of supervised ML models including logistic regression (LR), support vector machines (SVM), and ensemble methods such as random forest (RAF) and extra trees (39–42). This group of AI algorithms are often applied on time-independent tabular patient information. For textual, higher dimensional data, and grid like data types such as time series data and medical images, natural language processing (NLP), deep learning, convolutional neural networks (CNNs), and recurrent neural networks (RNNs) models are widely applied (25, 43, 44). It is quite common in this domain that basic classifiers such as LR and decision tree based methods are applied to simplified representations of datasets to provide baselines for comparison to more sophisticated methods (3, 14).

*Logistic regression*: As a supervised ML algorithm, logistic regression (LR) (45) is a predictive model leveraging the concept of probability to solve binary classification problems. Fundamentally, LR is a linear regression model with a special type of activation function, the so-called sigmoid function or logistic function which, based on a given decision boundary, quantifies the probability of belonging to each of the binary labels.

*Support vector machines*: Support vector machines (SVMs) (46) is a supervised ML algorithm that aims to find the optimal hyperplane which separates data points in one, two, or multi-dimensional space, depending on the complexity of the feature space. To maximize the probability of true classification of unseen data points, the chosen hyperplane has to expose the maximum possible distance, i.e., margin, between the data points of different classes, increasing the impact of the data points locating nearest to the hyperplane (i.e., support vectors).

*Decision trees and ensemble algorithms*: Decision trees employ tree-structured flowcharts of decisions based on the values of the input features to solve classification problems (47). At each node of such trees, a decision is made based on a single feature whether to make the final prediction or make another decision based on another feature. The leaves of a decision tree are the target labels. Ensemble algorithms such as random forest (RAF) (48) apply different randomized groups of decision trees, denoted as ensembles of trees, as well as different bootstrapping mechanisms to come up with the final decision on the target labels.

*Gradient boosting and categorical boosting*: Gradient boosting, which is used for classification and regression tasks, draws predictions as ensembles of some weak learners, mostly decision trees or random forests (49). When it comes to the analysis of categorical data, categorical boosting or CatBoost algorithm outperforms other gradient boosting methods (50).

*Recurrent neural networks*: In contrast to conventional feed-forward neural network models which are mostly used for processing time-independent datasets, RNNs are well-suited to extract non-linear interdependencies in temporal and longitudinal data as they are capable of processing sequential information, taking advantage of the notion of hidden states *h*. In such a model, at each timestamp *t,* the input data is processed alongside the information which was processed in the previous timestamp *t-1* (51). Also, for patient monitoring, a variety of RNN-based models such as long short-term memory (LSTM) and gated recurrent unit (GRU) are commonly applied.

First introduced by Hochreiter and Schmidhuber in 1997, LSTM (52) aims at identifying both short-term and long-term dependencies in the sequential data such as clinical time series data. LSTMs consist of cells with input, output, and forget gates which regulate the flow of information to remember values over arbitrary time intervals.

*Natural language processing*: When it comes to automated processing of textual patient data, such as electronic health records (EHRs), natural language processing (NLP) comes into action. NLP [Allen2003] denotes the set of AI based approaches which are capable of identifying underlying patterns in the textual data, hence understanding human languages. Taking the examples of EHRs and temporal textual patient information stored in medical databases such as Medical Information Mart for Intensive Care (MIMIC) (53, 54), clinical and medical domains also take advantage of NLP (15).

### 2.2. Established conventional scoring systems used in critical care

Alongside continuous monitoring of patients by the intensivists and medical staff during patients stays at ICUs, several scoring systems are widely used in critical care units to monitor and manage patients states such as the acute physiologic and chronic health evaluation (APACHE), the sequential organ failure assessment (SOFA), and the mortality prediction model (MPM) (55–57). Such scoring systems become handy in studies which aim at analyzing emerging AI methods for clinical decision making as they provide established baselines for comparison.

*Mortality Prediction Models (MPMs)* (56) *and APACHEs* (55) are mathematical models that estimate the probability of death for critically ill patients in ICUs based on patient data such as demographics, diagnoses, and physiological measurements. Each of MPM and APACHE use a different set of variables and algorithms to predict mortality risk. These models are useful in guiding clinical decision-making, evaluating ICU performance, and identifying risk factors for mortality. However, they have limitations and should be used alongside clinical judgment as they are not designed to replace it or provide definitive prognoses. The accuracy of MPMs may vary depending on the patient population and the specific model used, and they should be validated and calibrated before use in clinical practice.

*Sequential Organ Failure Assessment (SOFA)* (57) is a scoring system used to track the progression of organ dysfunction in critically ill patients in the intensive care units. It is based on the evaluation of six organ systems: respiratory, cardiovascular, hepatic, renal, coagulation, and neurological, with the score ranging from 0 to 4 for each organ system, and higher scores indicating greater dysfunction. The total SOFA score is the sum of the scores for all six organ systems, ranging from 0 to 24, and is calculated daily for each patient in the ICU. SOFA score is often used in clinical research and quality improvement initiatives in ICUs, and it has been shown to be a useful predictor of mortality in critically ill patients.

### 2.3. Medical data modalities for intensive patient care

From a general perspective, one can subdivide medical data modalities into the following subgroups: structured data (with and without timestamp) and unstructured data such as medical image modalities and electronic health records (EHR). Like other fields of data science, numerical tabular information such as patient demographic information (e.g., age and weight) can be used to form feature vectors for AI- and ML-based methods. In case of time-dependent measurements such as lab values and vital signs, the dimension of time (i.e., timestamp) should be integrated in the corresponding analysis pipeline, hence the clinical time series data. This section provides a brief overview of different data modalities used in the scope of this systematic review.

Numerous kinds of data in diverse modalities are processed by medical experts and intelligent systems for patient monitoring in ICUs. Clinical time series and electrocardiograms (ECGs) are among the most common types of data applied in this domain. Furthermore, open access databases facilitate objective performance analyses of the implemented AI methods.

*Clinical time series data*: Continuous patient monitoring leads to a magnitude of measurements captured and stored at discrete timestamps. Regardless of the disease type, a variety of temporal datasets such as Electronic health records (EHR), lab values, vital signs, diagnoses and treatments records can be used for patient monitoring (58).

*Electrocardiograms (ECGs)*: First invented by William Eindhoven in 1902, electrocardiograms (ECGs) (59) are recorded non-invasively from the patient’s body surface and are used to represent the heart’s electrical activity. ECGs are widely applied for diagnosing heart complications also in cardiac ICUs.

#### 2.3.1. Open access datasets

Ensuring that methodology can be replicated is a key consideration in data science, which typically necessitates the sharing of data. However, in the medical and clinical field, there are often additional ethical limitations and considerations when it comes to sharing patient data, which is considered highly sensitive and confidential. These ethical concerns must be balanced with the need for reproducibility in research. This highlights the importance of open access datasets for medical and clinical research. This subsection briefly introduces some of the most applied publicly available datasets for intensive patient care.

One of the majorly used information platforms in biomedical research and education is PhysioNet which offers free access to large collections of physiological and clinical data and related open-source software, and educational tutorials (60). Among the recently published extensive clinical data collections that are present in PhysioNet, datasets of High time-Resolution ICU Dataset (HiRID) (61), Medical Information Mart for Intensive Care (MIMIC-II, MIMIC-III and MIMIC-IV) (53, 54, 62), and eiCU (63) are the ones majorly used for studies about intensive care units.

MIMIC is a public database of de-identified electronic health records of over 60,000 adult patients admitted to the intensive care units at the Beth Israel Deaconess Medical Center. It contains information on demographics, diagnoses, laboratory tests, medications, and clinical notes collected from various sources such as bedside monitors, clinical documentation, and hospital information systems. The database has been widely used in clinical research and machine learning applications to develop predictive models, identify risk factors, and improve clinical outcomes. Access to the database requires an application process and approval from the Institutional Review Board at BIDMC, but it is publicly available through PhysioNet, a repository of physiological data and clinical information maintained by MIT.

Intensive care units (ICU) are a prominent source of time series data, as the nature of intensive care usually requires close and regular monitoring of patients and thereby produce a high density of measurements. Instances of time-dependent measurement data that can be found in publicly available ICU datasets include time-stamped nurse-verified physiological measurements such as hourly documentation of heart rate, arterial blood pressure, or respiratory rate. Other examples include documented progress notes by care providers, continuous intravenous drip medications, and fluid balances (53).

### 2.4. Interpretability and explainability of AI in healthcare

Usually, in intensive patient care, the mission of AI systems is to provide risk estimates and assist in decisions by providing predictions, which then need to be understood, interpreted and validated by clinicians. To assess the trustworthiness, the AI developers together with clinicians have different sorts of higher-order evidence at hand (64). Most importantly, as identified by related work (24) and discussed in some of the selected manuscripts (25, 65–67), before an AI system is being implemented in clinical settings, it is being technically and clinically validated. The validation yields evidence of a system’s accuracy and reliability through a standard procedure. Besides these evaluations, it is important to transfer the knowledge about what the AI system has focused its attention on through some *post hoc* explanations. This AI transparency is crucial in medical AI, especially in the use case of patient monitoring (68). Transparency refers to algorithmic procedures that make the inner workings of a ‘black box’ algorithm interpretable to humans (69). Another factor is traceability that intersects with the concepts of method and results in reproducibility and replicability of underlying data analysis. Covering these aspects relates to providing sufficient detail about procedures and data so that the same procedures could be exactly repeated. Auditability of AI shapes itself more and more as a necessary tool in achieving innovation in a secure, transparent way.

To interpret decisions made by AI models with deep architectures and to cope with their ‘black box’ nature, recursive feature elimination (RFE) and SHapley Additive exPlanations (SHAP) methods are commonly applied also in the medical AI domain. RFE takes an ML classifier and the desired number of features as input and starts from the entire input feature set. Then at each recursion step, the features are ranked based on an importance metric and the least relevant variables are removed. This procedure continues until the desired number of features are chosen (70). Inspired by game theory, SHAP is used to explain the output of any machine learning model by connecting optimal credit allocation with local explanations, assigning each input feature an importance value for a particular prediction (71). Nevertheless, the explainability provided by most of conventional methods such as RFE and SHAP is rather located on model level and addresses understanding of how a model derives a certain result, lacking the semantic context which is required for providing human-understandable explanations. In medical applications, the quest for explainability is usually motivated by medical semantic understanding, thus explainability on e.g., syndrome level which is the language of physicians (72).

## 3. Methods

### 3.1. Search strategy and screening

We followed the preferred reporting items for systematic reviews and meta-analyses (PRISMA) (32) and the population, intervention, comparator, outcome, and study design (PICOS) (33) guidelines. However, as meta-analysis was not originally intended for this study, we only followed the parts of PRISMA that only apply to systematic reviews. As this had led to a group of studies covering a diverse selection of datasets and algorithms, a comprehensive meta-analysis was not feasible. From the PubMed and Google Scholar databases, the following keywords are searched: (“artificial intelligence” OR “AI” OR “machine learning” OR “ML”) AND (“ICU” OR “intensive care” OR “intensive care unit” OR “intermediate care unit” OR “IMC” OR “IMU” OR “patient monitoring”) AND (“cardiovascular” OR “cardiac”). Moreover, a subjective literature research according to most relevant related studies complement results of the search engine queries. The publications dated from January 2018 to August 2022.

In the screening phase, original studies focusing on clinical decision support for adult subjects (age ≥17 years) visiting cardiovascular ICUs were analyzed. Thus, studies focusing on pediatric cohorts and review articles were removed from the results of search in the screening process. The summary of PICOS scheme containing the inclusion as well as exclusion criteria is outlined in Table 1.

**Table 1:** Population, intervention, comparator, outcome, and study design (PICOS) criteria for the systematic review.

Parameter	Inclusion criteria	Exclusion criteria
Population	Adults (age ≥ 17) Patients admitted to cardiovascular ICU	Age < 17 No cardiovascular patients No ICU patients
Intervention	Any	No restriction
Comparator	At least one AI/ML algorithm At least one control group	No AI/ML algorithm No control group
Outcomes	Any	No restriction
Study designs	Retrospective, prospective, or ambispective data analysis Hold enough data scientific significance Hold enough medical relevance	No proper statistical analysis significance No proper cross-validation No enough medical relevance

### 3.2. Quality assessment, selection criteria, and risk of bias assessment

All the papers collected as results of search engine queries were assessed whether they held enough significance and relevance from both data science and medical points of view. First, each of the papers underwent qualitative reviews by two independent reviewers which were selected randomly from a group of reviewers with data science and AI background. In case of agreement about selecting the manuscript between the two assigned reviewers, the manuscript would be short-listed or eliminated from the systematic review accordingly. On the contrary, in case of a mismatch between the assessments carried out by the first two reviewers, a third reviewer with higher qualification would decide whether to select or reject the manuscript. Consecutively, the selected papers underwent another assessment step by a group of medical experts whether they fit within the scope of this study: patient monitoring in cardiovascular ICUs. The technical criteria to assess the manuscripts qualitatively include proper research concept, representative train/test cohorts, and proper cross-validation either within the dataset or against external cohorts.

To visualize the risk of bias assessment results, the robvis package (73) is used. As the criteria for risk of bias, the following seven items have been considered: reasonable cohort size (D1), proper cross-validation (D2), external validation set (D3), blinding of participants and personnel (D4), blinding of outcome assessment (D5), incomplete outcome data (D6), and selective reporting (D7). To account for subjectivity, the bias assessment was conducted with the same approach as for the study selection, i.e., with random assignments to two reviewers followed by a final validation by a third expert.

## 4. Results

In this section, the results of the systematic review are elaborated. First, a summary of the screening step is given followed by narrative reviews of the selected papers. Afterwards, a comprehensive analysis of the papers is provided which comprise a risk of bias analysis and assessments of studies outcomes, used datasets, and applied algorithms. Furthermore, if existing, relevant discussions on the integration of AI in cardiovascular ICUs are reported.

### 4.1. Study selection

**Figure 1:**
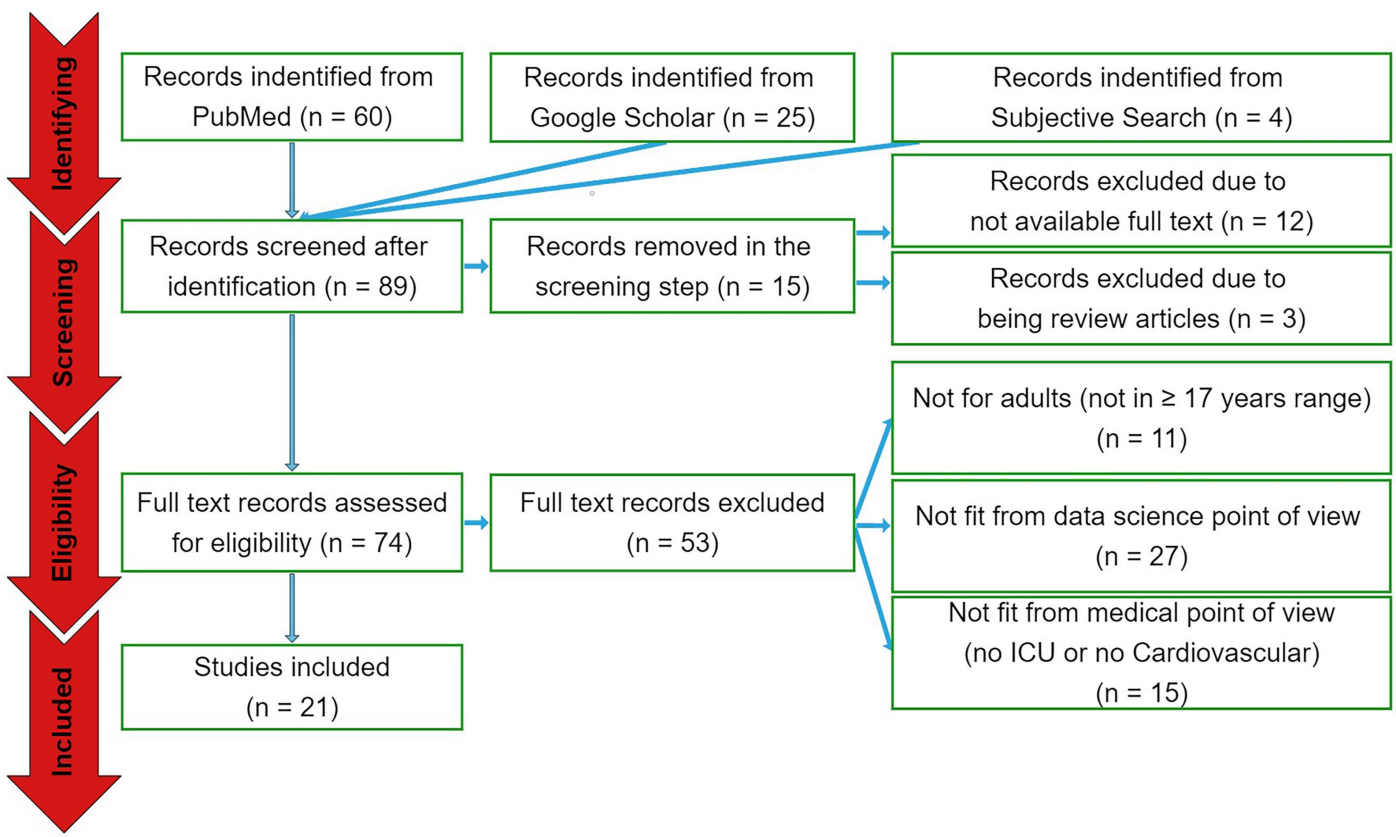
The PRISMA diagram. From a total of 89 papers identified by the search queries from the three sources, 15 and 53 papers were excluded in the screening and eligibility assessment phases, respectively. Accordingly, 21 papers were included to be reported.

The search engine queries have resulted in 89 papers in total. Out of these papers, 60 were from PubMed database and 25 were from Google Scholar. Another four papers were selected from subjective literature research from most relevant related articles. In the screening phase, 12 papers were excluded due to not available full text and three studies were excluded because of being review articles. In the eligibility assessment step, 11 papers were eliminated as they analyzed non-adult cohorts, 27 studies were excluded as considered not to be of proper significance from data science point of view, and 15 papers eliminated because they did not particularly focus on cardiovascular ICU cohorts (see Figure 1). As a result, 21 papers have been selected for the qualitative and quantitative analyses.

### 4.2. Summary of the included studies

Table 2 provides a summary of the important contents of the 21 included papers. This subsection presents a narrative review of these studies.

**Table 2:** The summary of the included studies. The most important contents of the 21 studies are summarized.

Study	Population	Study designs	Predicted outcome(s)	Data type(s)	Method(s)	Main contribution(s)	Identified challenge(s) towards integration of AI in practice
Zhao et al. (65)	16,189 adult (age > 18) patients from MIMIC-IV	Retrospective training, prospective validation	Extubation failure	Clinical time series (MIMIC-IV and domestic)	Categorical boosting with SHAP and RFE	Well-performing AI model (up to 0.83 AUROC), increased interpretability, open access UI for model validation	Interpretability, dataset shift problem
Jentzer et al. (66)	11,266 adult (Mean age 68 ± 15 years) patients from Mayo Clinic ICU	Retrospective data analysis	Mortality risk	Numerical clinical data extracted from ECGs (domestic)	Multivariate logistic regression	Well-performing AI model (up to 0.83 AUROC)	Interpretability
Gandin et al. (74)	10,616 patients from MIMIC III	Retrospective data analysis	Mortality risk	EHR (MIMIC-III)	RNN (LSTM with attention layer)	Well-performing AI model (up to 0.79 AUROC), attention layer to increase the interpretability of LSTM	Interpretability and reliability
Andersson et al. (67)	932 adult (age ≥ 18) patients from 36 ICUs across Europe and Australia	Retrospective data analysis	Neurological outcome following out-of-hospital cardiac arrest (OHCA)	Clinical variables and biomarkers (domestic-multicenter)	ANN with SHAP	Reliable AI model (up to 0.94 AUROC) using cumulative clinical data from first 3 days of ICU stay	Generalizability, effect of outliers
Parsi et al. (39)	53 patients from PhysioNet	Retrospective data analysis	Paroxysmal atrial fibrillation	ECG (PhysioNet)	SVM, k-NN, RF, MLP	High performance AI (up to 0.79 accuracy) on implantable defibrillator with low computation power	Low computational power on wearable and implantable devices
Yu et al. (40)	7,368 adult (age > 18) patients from MIMIC-III	Retrospective data analysis	4-year mortality risk after cardiac surgery	Clinical time series (MIMIC-III)	LR, ANN, Ada, NB, RF, etc. with RFE	Well-performing AI model (up to 0.80 AUROC), open access UI for model validation	Generalizability
Wang et al. (75)	929 adult (age > 18) patients from eICU-CRD	Retrospective training, prospective validation	Noninvasive ventilation (NIV) failure	Clinical time series (eICU-CRD and domestic)	Categorical boosting with RFE and SHAP	Well-performing AI model (up to 0.87 AUROC) applied to easily available clinical variables, open access UI for model validation	Generalizability, low specificity of AI predictions
Chen et al. (41)	1,439 adult (mean age 65.05 ± 12.53 years) patients from Cheng Hsin General Hospital	Retrospective data analysis	Ventilator weaning time	Non-time series clinical data (domestic)	LR, SVM, RF, ANN, XGBoost	Well-performing AI model (up to 0.88 AUROC), identify most simplified key parameters	Generalizability
Dutra et al. (76)	519 adult (age > 18, mean age, 74.87 ± 13.56 years) patients admitted to a Brazilian cardiac ICU	Ambispective data analysis	Mortality risk from heart failure with mid-range ejection fraction (EF)	Non-time series clinical data (domestic)	Cox, Kaplan–Meier, ElasticNet, survival tree	EF is not significantly correlated with mortality	Generalizability
Bodenes et al. (42)	540 adult patients admitted to Brest University Hospital’s cardiac ICU	Prospective data analysis	Mortality risk and heart rate variability (HRV)	Clinical time series (domestic)	k-NN, SVM, LR, decision trees	Low cost and efficient AI model for HRV analysis	Generalizability, interpretability, lack of standardized HRV measurement methods
Moazemi et al. (25)	11,513 patients from MIMIC-III and 502 from University Hospital Düsseldorf’s cardiac ICU (age ≥ 17)	Retrospective data analysis	ICU readmission	Clinical time series (MIMIC-III and domestic)	RNN (LSTM)	Well perforing AI (up to 0.82 AUROC), data-driven approach, validation with external cohort	Interpretability, dataset shift problem
Baral et al. (44)	7,611 patients (age > 15) from MIMIC-III cardiac ICUs	Retrospective data analysis	Cardiac arrest	Clinical time series (MIMIC-III)	Multi-layer perceptron (MLP), RNN (bidirectional LSTM)	Well-performing AI model (up to 0.94 AUROC) to reduce false alarm for cardiac arrest, improved model compared to normal LSTM	Generalizability
Qin et al. (43)	49,168 patients from MIMIC-III	Retrospective data analysis	Sepsis	Textual and structured clinical data (MIMIC-III)	NLP (BERT), Amazon Comprehend Medical for data processing, XGBoost (for classification)	Outperform PhysioNet’s sepsis prediction challenge winner (up to 0.89 AUROC)	Generalizability
Nanayakkara et al. (77)	Adult (age ≥ 17) septic patients from MIMIC-III	Retrospective data analysis	Sepsis treatment planning	Clinical time series (MIMIC-III)	RL	Introducing a novel physiology-driven recurrent autoencoder, highly interpretable, uncertainty quantification	Lack of standardization, how/when AI is considered safe enough for clinical routine
Zheng et al. (78)	1,362 critically ill COVID patients (mean age 69.7) from New York University Langone Health	Retrospective data analysis	Managing oxygen flow rate to reduce mortality risk	EHR (domestic)	RL	AI model to identify optimal personalized oxygen flow rate to reduce mortality rate	Generalizability
Peine et al. (79)	61,532 and 200,859 ICU stays of adult patients from MIMIC-III and eICU datasets	Retrospective data analysis	Optimization of mechanical ventilation to reduce mortality risk	Clinical time series (MIMIC-III and eICU)	RL	Introduce VentAI to dynamically optimize mechanical ventilation for individual patients	Generalizability, algorithm bias, missing/false data
Akrivos et al. (80)	162 adult patients (18 < age < 90 on) from MIMIC-II	Retrospective data analysis	Cardiac arrest	Transformed clinical time series (MIMIC-II)	integrated model of sequential contrast patterns using Multichannel Hidden Markov Model	High sensitivity (with the average of 0.78) and specificity to identify high risk patients	False positive rate in classification results
Aushev et al. (81)	75 adult (age > 18)patients from ShockOmics European database	Retrospective data analysis	Mortality due to septic and cardiogenic shock	ECG (ShockOmics Dataset)	SVM, Random Forest, RFE, Bayesian networks	Apply feature selection to identify the most relevant predictors of mortality due to septic and cardiogenic shock using ECG with high certainty (up to 0.84 AUROC)	–
Kim et al. (82)	29,181 adult (age > 18) ICU patients from Yonsei Health System (Severance and Gangnam Severance Hospitals)	Retrospective data analysis	Acute respiratory failure and cardiac arrest	Time series (domestic)	Deep Learning (LSTM)	Introduce FAST-PACE for preparing immediate intervention in emergency situations, outperforming some established scoring systems (e.g., SOFA) (up to 0.88 AUROC)	Lack of relevant input data to AI models, lack of external validation, imbalanced datasets, lack of real time measurements of vital signs
Meyer et al. (83)	11,492 ICU stays from 9,269 adult (age ≥ 18) patients from a German cardiovascular tertiary care center	Retrospective data analysis	Mortality, renal failure, postoperative bleeding leading to operative revision	Time series (domestic)	Deep learning (RNN)	Predict severe complications after cardiothoracic surgery with a higher certainty (up to 0.96 AUROC), validation against MIMIC-III dataset	Dataset shift, biased data, generalizability, transparency and interpretability of AI decision making
Yoon et al. (84)	2,809 Adult (age > 18) patients from MIMIC-II	Retrospective data analysis	Tachycardia as a surrogate for cardiorespiratory instability (CRI)	Vital signs time series (MIMIC-II)	Regularized logistic regression (LR), Random Forest	Developed a risk score for predicting tachycardia episodes, AI model with high accuracy (up to 0.86 AUROC)	Timestamp mismatching and data sparsity, specificity of predictions, lack of external validation

Zhao et al. (65) integrated a categorical boosting ML model to predict extubation failure resulting in in-hospital or 90-day mortality in patients visiting ICUs. To train their model, they used clinical time series data from the MIMIC-IV database. For the test purposes, they applied an external data set. To identify the most important predictive factors, they applied RFE and SHAP methods. Their results suggest that critically ill patients might benefit from AI assisted mechanical ventilation. They also provide an UI for model validation which is freely accessible online. They mention interpretability and inconsistency in train and test datasets as the most critical challenges towards integrating AI in clinical practice.

Jentzer et al. (66) used multivariate logistic regression on numerical clinical variables extracted from ECGs from their own facilities to quantify mortality risk due to left ventricle systolic dysfunction in patients staying at ICUs. Their findings suggest the relevance of the AI-driven methodology for the quantification of cardiac patients’ survival potential and identify lack of explainability as a challenge to be handled before it can be integrated in prognostic pipelines.

Gandin et al. (74) investigated the interpretability of an RNN model with long short-term memory (LSTM) architecture as used for survival prediction in a cohort of patients visiting cardiovascular ICUs. They analyzed the MIMIC-III dataset for both training and test purposes. The results of their study demonstrate that incorporating an attention layer into the LSTM model can enhance the interpretability of the AI model’s decisions, leading to greater reliability in AI decision making.

Andersson et al. (67) took advantage of artificial neural networks (ANNs) to anticipate neurological outcomes due to out-of-hospital cardiac arrest (OHCA). They analyzed clinical variables and biomarkers from a cohort of patients from their own hospital and used SHAP method for identifying the most relevant factors. They showed that the clinical parameters captured in the first 3 days of ICU stay contribute to OHCA prognostication. Although their results suggest reliable predictions, they insist on external validation with larger cohorts to assess generalizability of their methods.

Parsi et al. (39) took advantage of supervised machine learning methods such as support vector machines (SVM) to analyze data extracted from ECGs to predict paroxysmal atrial fibrillation in ICU patients with high accuracy. For their training and test, they applied open access data from the atrial fibrillation prediction database (AFPDB) of PhysioNet. Their primary contribution involves integrating an AI model with high performance onto implantable devices with low computational power.

Yu et al. (40) evaluated several ML models including logistic regression, random forest, and adaptive boosting (Ada) as applied to clinical time series data (from MIMIC-III database) for the prediction of long-term survival of patients after cardiac surgery, highlighting the significance of Ada model. As the generalizability plays an important role in integration of AI-assisted methods, they also provide a freely accessible online platform for the validation of their model against external sets of data.

To predict noninvasive ventilation (NIV) failure in cardiac ICU patients, Wang et al. (75) took advantage of categorical boosting alongside RFE and SHAP methods for analyzing most important factors among clinical time series data. They used open access data from the eICU-CRD database for training and data from their own hospital for test purposes. They have shown the relevance of the AI model and provide an online tool for model validation, while identifying lower specificity in predictions of AI as the most challenging issue which limits generalizability of their findings.

Chen et al. (41) analyzed different supervised ML classifiers (including logistic regression, SVM, random forest, artificial neural networks and XGBoost) for the task of predicting ventilator weaning in the next 24-h time windows, given non-time series clinical data corresponding to a cohort of cardiac ICU stays in their facilities. Their key finding is that ventilator weaning can be anticipated using a limited number of clinical factors such as expiratory minute ventilation, expiratory tidal volume, ventilation rate set, and heart rate. As they only applied data from their own center, generalizability of their findings remains in question.

Dutra et al. (76) applied a variety of statistical and ML methods including Cox and Kaplan–Meier estimators as well as ElasticNet (85) and survival trees to quantify mortality risks of ICU patients due to heart failure with mid-range ejection fraction (EF). Their findings suggest that there is no significant correlation between EF and survival probability of the patients. As they only analyzed data from a single center, their findings are subject to bias, hence the need for follow-up generalizability assessments.

Bodenes et al. (42) applied and compared AI classifiers such as k-NN, SVM, and decision trees to predict survival of the ICU patients due to heart rate variability (HRV). They analyzed clinical time series from a single center and proposed a low cost and efficient model for HRV analysis. However, their findings are subject to further assessments against external data cohorts. They also identified the lack of global standardization of HRV measurement methods and interpretability of AI models as limitations to overcome in the future.

Moazemi et al. (25) evaluated two alternative long short-term memory (LSTM)-based models to predict readmission risks in cohorts of cardiovascular ICU patients, analyzing clinical time series data as well as patient level information. They used a cohort of cardiac ICU stays from MIMIC-III as well as a dataset from their own hospital for train and test purposes, respectively. Their findings highlight the benefit of RNN models in general, and the need for consistency in train and validation cohorts in particular. They further highlight the dataset shift problem and interpretability of deep learning models as critical future challenges for AI in CDS.

Baral et al. (44) applied multi-layer perceptrons (MLP) and bidirectional LSTM models for the prediction of cardiac arrest and have shown the superiority of the enhanced bidirectional model to the normal LSTM. They analyzed a cohort of data from MIMIC-III for both training and test purposes. Their proposed RNN model showed reasonable performance in predicting cardiac arrest, reducing the false alarm rate significantly. As they did not validate their model with external data, their findings are subject to further generalizability assessments.

Qin et al. (43) applied Bidirectional Encoder Representations from Transformers (BERT) (86) and Amazon Comprehend Medical techniques (as natural language processing (NLP) approaches) to process textual data and XGBoost method to classify patients with high risk of sepsis. They leveraged open access and structured clinical data from the MIMIC-III database for training and test. Their proposed pipeline outperformed the winner of PhysioNet challenge for sepsis prediction in 2019 which had applied XGBoost and Bayesian optimization without processing textual data (87). However, their findings lack validation against independent external cohorts, hence the generalizability issue.

Nanayakkara et al. (77) took advantage of reinforcement learning approaches to introduce a novel recurrent autoencoder for the task of sepsis treatment planning. They used clinical time series data from the MIMIC-III database for their analysis which include interpretable uncertainty quantification of clinical factors. They further discussed the lack of globally agreed standards in the assessments of safeness of AI methodologies as one of the most critical challenges in the field.

Zeng et al. (78) also applied reinforcement learning methodologies to quantify the optimal personalized oxygen flow rate to minimize the risk of mortality in cardiac ICU patients. To this end, they analyzed electronic health record (EHR) data from cardiovascular patients’ stays at their hospital in a single center study. Thus, their findings might be subject to future external validation.

In another study leveraging reinforcement learning methodologies, Peine et al. (79) introduced VentAI, an RL based pipeline for personalized optimization of mechanical ventilation in patients staying at cardiovascular ICUs. They analyzed clinical time series data from two open access databases (MIMIC-III and eICU) and identified generalizability, bias in AI algorithms, and missing and false entries in the measured clinical parameters as the most important challenges towards integration of AI in clinical practice.

Applying regularized logistic regression and random forest algorithms to vital signs from MIMIC-II dataset, Yoon et al. (84) suggest that predicting tachycardia could increase clinical awareness of a higher risk of future hypotension and subsequently other forms of cardiorespiratory instability (CRI). But they did not directly compare their model to conventional scoring systems or conduct validation studies against independent sets of data.

Meyer et al. (83) applied a deep recurrent model to analyze time series data for the task of predicting severe complications in critical care units after cardiovascular surgery such as mortality, renal failure, and postoperative bleeding leading to operative revision. Their model outperforms clinical reference tools and is available to be integrated in EHR systems. They further validate the performance of their model which is trained using domestic data against external data from the MIMIC-III database and highlight the importance of generalizability and interpretability of AI methods in clinical practice.

Kim et al. (82) introduced Feasible Artificial Intelligence with Simple Trajectories for Predicting Adverse Catastrophic Events (FAST-PACE), an LSTM model to process clinical time series data, to predict events of acute respiratory failure and cardiac arrest. They fit their model using a domestic cohort of data and show the superiority of their model compared to some established scoring systems such as sequential organ failure assessment (SOFA) and mortality prediction model (MPM). Their findings further identify lack of external validation and inconsistencies in real time measurement schemes in critical care units as some limitations of data-driven approaches towards clinical decision making.

Aushev et al. (81) applied different feature selection techniques such as recursive feature elimination (RFE) in combination with SVM and random forest classifiers to identify most relevant features that could predict mortality due to shock in the intensive care unit. To this end, they analysed ECG data from ShockOmics dataset as part of an Europe funded project. As their patient cohort with 75 subjects is relatively small, their findings might be subject to further assessment.

Akrivos et al. (80) took advantage of the MIMIC-II dataset to integrate a model of sequential contrast patterns using the Multichannel Hidden Markov Model which is able to predict cardiac arrest in cardiovascular ICUs. Their approach takes advantage of clinical time series data after transforming them to sequential patterns. Their model achieves high performance, while suffering from a relatively low false positive rate in classifier predictions. This identifies rooms for follow-up studies including data from independent databases.

### 4.3. Risk of bias assessment

**Figure 2:**
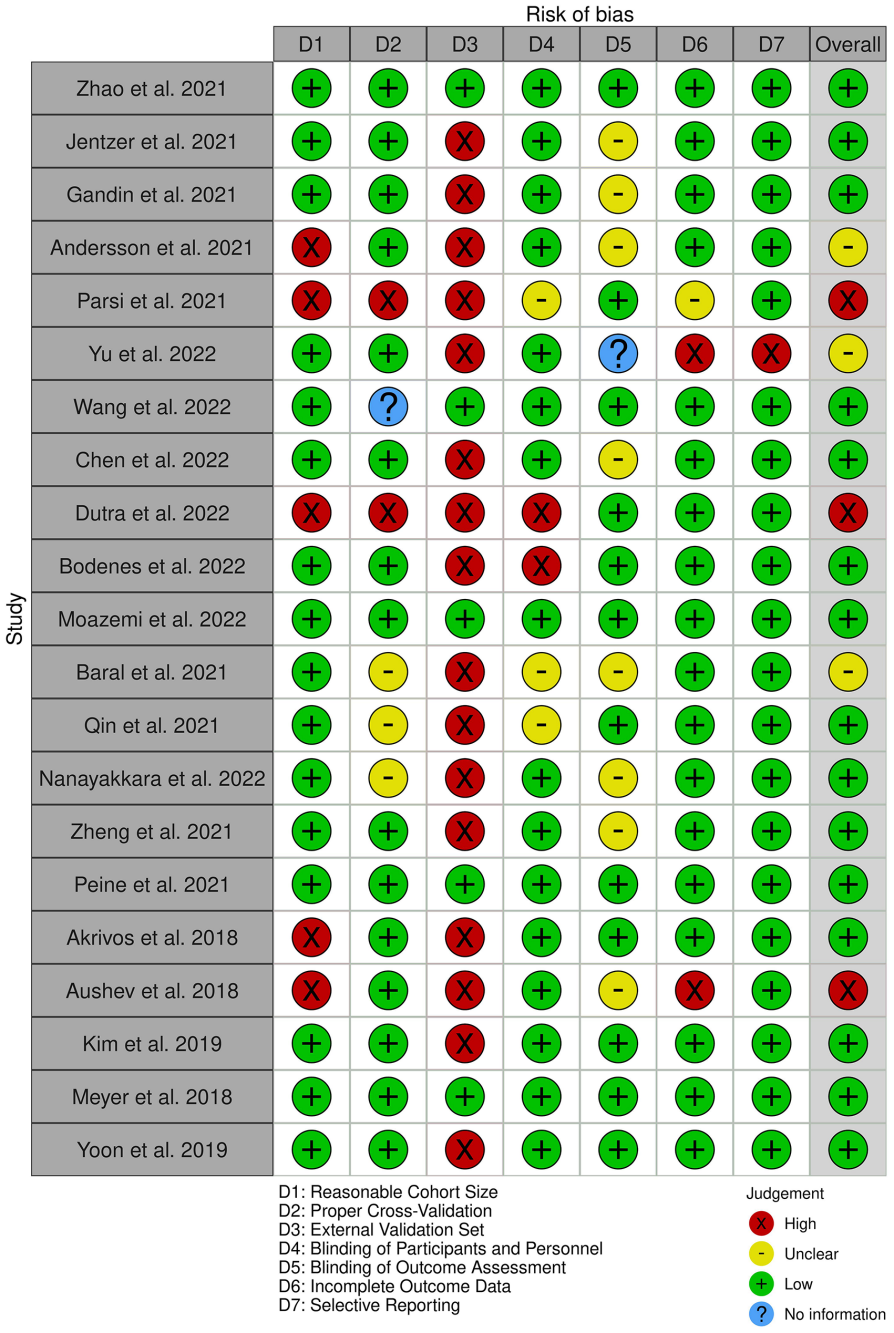
The risk of bias diagram for the selected studies. Each row corresponds to a selected study. The columns D1–D7 correspond to different risk criteria. The subjective judgements are color-coded as explained in the legend. The final column represents the overall judgement for the corresponding study.

**Figure 3:**
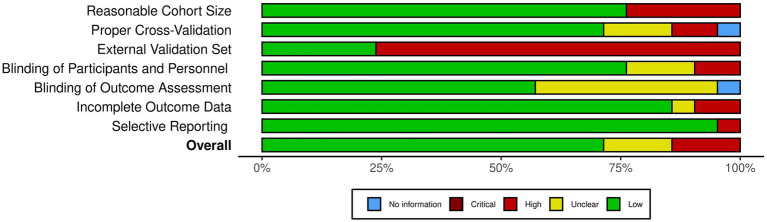
The summary of the risk of bias analysis. Each bar chart corresponds to one criteria of bias, stacked along the Y axis. The X axis quantifies the percentage of the studies with the corresponding color-coded subjective assessment as explained in the legend.

Figure 2 provides an overview of the risk of bias analysis results. Most of the studies conducted proper cross-validation methods. However, only five studies used independent external datasets for the validation of their models (Figure 3), which identifies lack of generalizability as a common issue towards integration of AI methodologies across different research groups and medical centers.

### 4.4. Studies’ outcomes

**Figure 4:**
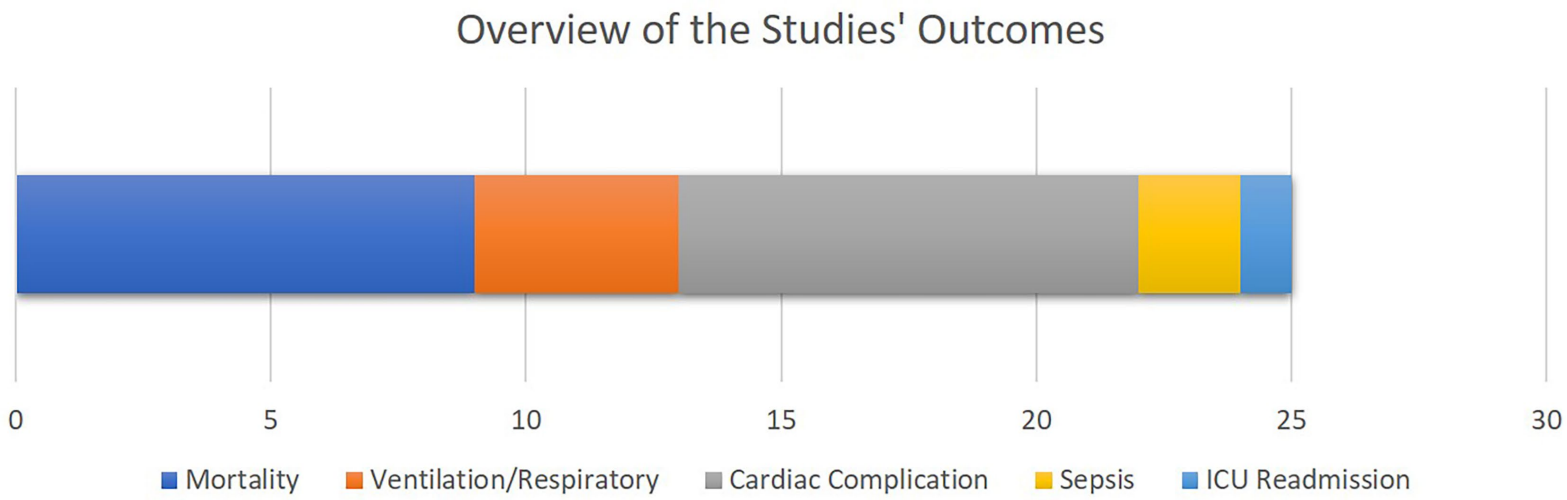
The overview of the outcomes of the selected studies. The bar chart shows how frequent each study outcome has been, with the X axis quantifying the number of studies. Note that some studies analyzed multiple outcomes.

As illustrated in Figure 4, mortality as well as cardiac, sepsis and respiratory complications rank amongst the most common clinical outcomes analyzed by the selected literature. This is justified as most of the patients visiting cardiovascular ICUs have had cardiac surgeries beforehand or are subject to higher cardiac and respiratory complications.

### 4.5. Analyzed data types

**Figure 5:**
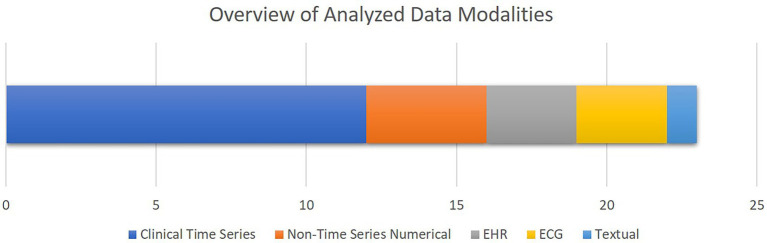
The overview of the data modalities analyzed in the selected studies. The bar chart shows how frequent each data modality has been, with the X axis quantifying the number of studies. Note that some studies analyzed multiple data modalities.

Figure 5 shows an overview of the data modalities analyzed in the selected papers. Clinical time series is the most common group, while EHR and textual data are the least common groups. Moreover, as presented in Table 2, 13 studies out of 21 selected studies utilized open access datasets with 10 studies using different versions of MIMIC database either for training or validation purposes.

### 4.6. AI algorithms and models

**Figure 6:**
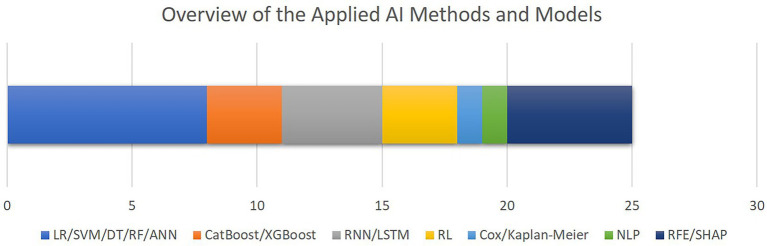
The overview of the AI methods and models applied for outcome prediction or interpretability. The bar chart shows how frequent each AI method has been, with the X axis quantifying the number of studies. Note that some studies applied multiple algorithms or methods (LR, logistic regression; SVM, support vector machine; DT, decision trees; RF, random forest; ANN, artificial neural networks; CatBoos, categorical boosting; XGBoost, extreme gradient boosting; RNN, recurrent neural networks; LSTM, long short-term memory; RL, reinforcement learning; NLP, natural language processing; RFE, recursive feature elimination; SHAP, SHapley Additive exPlanations).

Figure 6 outlines the AI methods for model development and interpretation of the models’ decisions as utilized by the included studies. The most common group of algorithms are linear or decision tree-based methods, followed by recurrent models. Only five studies included feature selection or explainable AI methods. Although the high level of diversity in the datasets and algorithms which are utilized in the selected papers hinders us from conducting comprehensive performance meta-analysis, as outlined in Table 2, area under the receiver operating characteristics curve (AUROC) ranging from 79 to 96% throughout the entire cohort of papers, is the most commonly reported metrics item.

### 4.7. Concerns towards integration of AI in clinical routine

**Figure 7:**
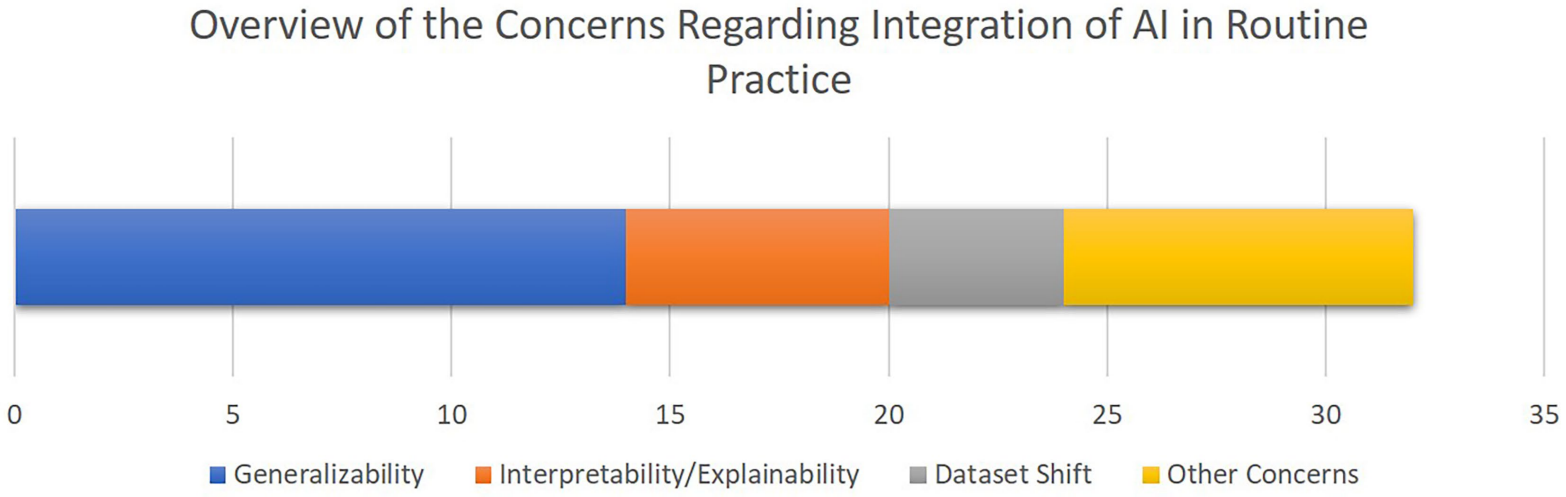
The overview of the concerns towards integration of AI-driven decision support tools in clinical routines as discussed in the selected studies. The bar chart shows how frequent each concern has been, with the X axis quantifying the number of studies. Note that some studies mentioned multiple concerns.

Figure 7 provides an overview of the concerns and limitations for the integration of AI for CDS in cardiac ICUs as discussed in the included papers, highlighting generalizability, interpretability, and dataset shift as the most central issues.

## 5. Discussion

Conventionally, patients visiting different care units undergo continuous examinations and interventions during their stays at the corresponding units. Thus, the physicians and medical staff are required to proactively monitor all the patients’ critical signs and examination results regardless of their types and frequencies. In particular, for cardiovascular patients who are subject to higher complication rates and longer stays at intensive care units (ICUs) (4, 14), the increasing amounts of propagated and interconnected health-related factors captured along the patients’ stays expose challenges towards taking appropriate and timely decisive actions for the physicians. These challenges are signified as many of the sources of multimodal temporal data used to make diagnostic or prognostic decisions, such as EHR extracted laboratory variables and vital signs, might be non-linearly correlated. Therefore, to assist the physicians and to complement their decision-making routines, there is an evolving need for appropriate clinical decision support systems (CDSS) leveraging modern AI-driven methodologies which are capable of investigating and identifying non-linear correlations in the multimodal patient data.

Advancements in AI are taking place continuously. Their presence in medicine is ever growing, and they could soon be present in cardiac ICUs. AI has the ability to assist clinicians in diagnosing arrhythmias, as shown in Parsi et al. where they were able to detect atrial fibrillation with a sensitivity and specificity >96% (39). Atrial fibrillation is a very common complication post cardiac surgery, which if not recognized, can have a significant negative impact on a patient’s health. The sooner atrial fibrillation is detected and treated, the higher are the chances of conversion into sinus rhythm. Another role AI can play is predicting therapeutic outcomes and thereby helping plan for further treatment. In the paper by Andersson et al. the authors showed their ANN provided good prognostic accuracy in predicting neurological outcomes in comatose patients post out-of-hospital cardiac arrest (67). By having the capability to predict neurological outcomes, AI can help physicians decide whether further treatment would be beneficial for patients with neurological complications post cardiac arrest in the form of neurological rehabilitation for instance. Thus, it could help improve patient quality of life in those who would benefit, as well as filtering those who would not, thus ideally lowering the demand for neurological rehabilitation spots in clinics, which are already oversaturated with patients on waiting lists. Finally, AI is capable of optimizing and fine tuning therapies, as shown in Peine et al. concluding AI was capable of delivering high performance optimization of mechanical ventilation in critical care, sometimes even exceeding physicians in comparison (79), and in Zheng et al. where AI was able to calculate the optimal oxygen therapy in COVID-19 patients, which was shown to be less on average than the amount recommended by physicians (78). This goes to show how AI is capable of improving general treatment and patient outcomes in ICUs while at the same time reducing the usage of costly materials, resources and services.

As illustrated in Figures 2, 3, our risk of bias analysis shows that most of the studies pass the criteria regarding blinding of the assessments and reporting bias. However, the findings revealed rooms for further consideration of universal validation guidelines, highlighting the lack of validation against external data cohorts. Thus, compared to conventional risk of bias criteria, we included three extra criteria (D1–D3) which address data-driven aspects of bias considering cohort size, proper cross-validation, and external datasets for validation purposes. We believe, integrating these extra bias assessment criteria should be followed in systematic reviews in the medical AI field.

To provide an overview of the results of this systematic review, most of the selected studies focused on critical cardiac and respiratory complications resulting in mortality of patients visiting cardiac ICUs (Figure 4). To this end, as illustrated in Figure 5, numerical measurements (either singular or time-dependent) captured during patients’ stays at ICUs are extensively used for model training and evaluation in most of the studies, while textual data are the least used modality in this regard. Consecutively, depending on the input data, suitable AI-methods are utilized for model development. As shown in Figure 6, supervised ML classifiers such as SVM and random forest alongside XGBoost and CatBoost and reinforcement learning (RL) are the most common methods. Moreover, when it comes to analyzing clinical time series data and textual data, recurrent neural networks (RNNs) and natural language processing (NLP) come to action, respectively. For the special case of integrating NLP for processing textual health records, the lack of systematic guidelines for reporting EHRs becomes critical when no persistent vocabulary exists, especially for the non-English speaking centers for which less data is available for training and validation purposes.

Our findings further highlight the importance of utilizing open access datasets to provide AI-assisted clinical decision support in cardiovascular ICUs. While there are clear benefits to using open access datasets such as MIMIC in the field of critical care, it is important to consider the potential limitations of such datasets. Open access datasets may not fully capture the nuances of specific healthcare systems or populations in certain regions, which may impact the generalizability of the AI models trained on them. Therefore, researchers and clinicians should carefully evaluate the suitability of open access datasets for their particular use case and consider supplementing them with domestic datasets if necessary. Nonetheless, open access datasets can facilitate collaboration and knowledge sharing, which are essential for advancing the field of AI-assisted clinical decision making. Also, open access datasets are often rigorously curated and annotated by experts, ensuring the data is of high quality and can be used reliably. On the other hand, domestic datasets may not have the same level of diversity and may be limited in size, leading to suboptimal AI models. Nevertheless, regardless of the fact that which kind of data is used to fit AI agents, a proper cross-validation scheme should be applied to account for generalizability.

As illustrated in the analysis results, logistic regression (LR), SVM, decision trees, random forests, neural networks, and recurrent deep learning models are all popular machine learning algorithms used for various tasks in the field. Each of these algorithms has its own strengths and weaknesses, and the choice of algorithm depends on the specific task at hand and the available data. Most of the time, LR, SVM, and often tree-based methods are used as baseline methods to complement other more complex methodologies such as deep or recurrent neural networks (RNNs). Furthermore, decision trees and random forests are good choices when dealing with small to medium-sized datasets that have both categorical and numerical features. They work well when the data has a clear and interpretable structure, and when the decision-making process can be represented as a sequence of simple if-then-else rules. Decision trees are also good when there is a need to explain the reasoning behind a model’s decision-making process. Neural networks, including deep learning models, are ideal for large and complex datasets with many features, such as image, speech, and text data. They are especially powerful when the relationships between input and output data are highly nonlinear and difficult to capture with simple models. However, neural networks can be computationally expensive to train and require a lot of data to generalize well. Recurrent deep learning models are a type of neural network that are well-suited for sequential and longitudinal data, such as time series, speech, and text data. They can capture long-term dependencies and patterns in the data and are especially useful when the output depends on past inputs. However, they can be more difficult to train than linear or tree-based models and require more specialized expertise. In summary, it’s important to evaluate the strengths and weaknesses of each machine learning algorithm carefully and select the one that is best suited to the specific needs.

The findings from the selected articles have shown the predictive potential of different AI approaches including RNNs and RL. While many of the included studies integrated supervised ML classifiers like SVMs or RNNs for continuous patient monitoring in cardiac ICUs, one general advantage reinforcement learning provides over other paradigms of ML is that this way of defining the problem allows RL to take into account long-term rewards. This characteristic makes it especially appealing for clinical applications since, in numerous healthcare issues, the response to treatment decisions is frequently delayed (88). Additionally, the exploration-exploitation approach shares similarities with the actual clinical setting, where treatment responses can be heterogeneous (89) and finding the optimal treatment regime can also be characterized by trade-offs between exploration and exploitation.

Based on the findings of the included literature, the most critical limitations towards integration of AI-driven methods in routine clinical decision making are generalizability and explainability issues. As illustrated in Figure 3, more than 75% of the studies lack validation against external datasets which highlights the lack of generalizability associated with their findings. Nevertheless, as presented in Table 2, only three of the 21 included studies provided open access web-based user interfaces to facilitate validating their models with external datasets. Although providing freely accessible tools for external validation should be marked as a benefit for novel AI tools, the lack of standardization of external validation schemes considering the high levels of privacy and confidentiality associated with medical data cohorts rank amongst the most important limitations towards integration of AI in clinical routines, especially in multicentric and federated scenarios (90).

Furthermore, despite all the promising achievements of AI in the medical domain, the medical experts are still responsible for patients’ lives. Therefore, to reduce the burden of responsibility and to provide further support, it is of critical importance to build trust in decisions made by the AI-assisted agents. As discussed in the related work (24), interpretability facilitated by explainable AI (XAI) best practices plays an important role to build further trust in AI in the medical domain. Although the authors of most of the reported articles recognize interpretability as a central issue in this domain, only five studies integrated methods such as RFE and SHAP to provide a level of transparency to complement their proposed models’ decisions (Figure 6). In a related work, Asan et al. (91) identified transparency, robustness, and fairness as the most important criteria to enhance trust when it comes to human-AI collaboration in the healthcare domain which is confirmed by our risk of bias analysis as well. This emphasizes the evolving need for extra efforts to identify and mitigate different sources of bias since the early stages of designing and developing AI models for the clinical and medical domains.

Another concern which affects the effective integration of AI methodologies in the healthcare domain is the certification of the established models and products upon proper evaluations and clinical trials. Although an increasing number of approved AI/ML products has been traceable since 2015 in the united states and Europe in domains such as radiology, related works urge for more transparency on the criteria for the approval of AI/ML-based products facilitated through publicly accessible databases from authorities such as the food and drug association (FDA) of united states of America (United States) and Conformité Européene (CE) of Europe (92). As an insightful example, Zanca et al. suggest some practical guidelines for the medical physicists (MPs) who conventionally act as responsible authorities to ensure safety and quality of emerging diagnostic and therapeutic technologies in healthcare. They empathize that MPs need to acquire enough knowledge about AI tools and how they conceptually differ from traditional medical software and hardware devices, because they often attribute higher levels of autonomy compared to traditional medical products (93).

The current study presents a comprehensive overview of the most widely used AI-related methodologies as reported in recent literature, which were selected in a systematic and objective manner. As a result, the majority of the methodology employed is based on modern machine learning solutions. However, as per some other studies such as Roller et al. (94), there is a suggestion to begin with simpler systems which make the use of explicit, structured knowledge such as guidelines, decision-making procedures, and thresholds which are commonly found in clinical environments. As our comprehensive analysis outlined, these often simpler “rule-based” processes have been mostly overlooked in the selected articles. This is an important concern which needs to be further addressed in follow-up studies.

As a limitation of current study, due to diverse datasets and algorithms used in the selected cohort of studies, it was not feasible to conduct comprehensive meta-analysis covering comparison of all the methods across all the databases. Nonetheless, we reported performance results from all the articles in Table 2. Although the results are not directly comparable with each other, area under the receiver operating characteristics curve (AUROC), ranging from 0.79 to 0.96, was the most universal performance metric across all the selected studies.

In this study, we included studies from PubMed and Google Scholar databases alongside additional papers chosen from subjective search queries within impactful related works. Also, we focused on the studies written in the English language. Thus, our findings might be biased with regard to the choices of search engines and text language and might not be fully comprehensive. However, we covered the application oriented, model-driven, and data-driven aspects of AI-assisted methodologies utilized for patient monitoring and medical intervention in cardiovascular ICUs, following the PRISMA (32) and medical AI life cycle (34) paradigms.

## 6. Conclusion and future work

*Technical conclusion*: Recent advancements in AI-driven methodologies in intensive patient monitoring open up new horizons for the integration of clinical decision support in practice. However, regardless of being totally automated or requiring an expert’s input or annotation, AI assisted methodologies for clinical decision support are meant to operate as a complementary aid to physicians and intensivists’ subjective decisions rather than acting in complete autonomy. To achieve this, certain limitations should be mitigated. Most importantly, to address the generalizability issue which has been highlighted by our findings to be a common source of bias, proper validation against independent unseen sets of data should be taken care of. This becomes more critical as the medical datasets attribute high levels of confidentiality, affecting multicentric and federated learning scenarios.

*Medical conclusion*: AI has the potential to simplify part of the decision making in intensive patient monitoring by reducing the burden of processing huge amounts of information available from different sources of vital signs and critical patient parameters. However, still efforts need to be made to enhance interpretability of state-of-the-art AI methods for clinicians. In addition, proper training and understandable insights should be provided for the medical staff to enhance the level of trust in AI decisions. Moreover, AI algorithms should be tested in prospective clinical trials similar to other new medical devices under observation of legal instances such as FDA in the United States and CE in Europe.

*Future work*: For the future, we plan to conduct studies on the integration of eXplainable AI (XAI) best practices for patient monitoring in cardiac ICUs, focusing on federated learning scenarios in which data from multiple hospitals are processed.

## Data availability statement

The original contributions presented in the study are included in the article/supplementary material, further inquiries can be directed to the corresponding author/s.

## Author contributions

SM, SV, MT, HA, and FS: conceptualization and study design. SM, SV, JL, PS, SK, RB, and BD: identification of papers through search engine queries. SM, SV, SK, and PS: technical review of searched papers. LC, HA, RAB, and AL: medical review of searched papers. JL, BD, RB, and PS: conducting visual analysis and preparing figures. SM, SV, PS, and LC: risk of bias analysis. SM, SV, PS, SK, BD, and RB: summarizing selected papers’ contents. SM, SV, MT, RAB, HA, and FS: narrative discussion on the findings. All authors contributed to the article and approved the submitted version.

## Funding

The project is funded by the German Federal Ministry of Education and Research (BMBF) under the grant number 16SV8601.

## Conflict of interest

The authors declare that the research was conducted in the absence of any commercial or financial relationships that could be construed as a potential conflict of interest.

## Publisher’s note

All claims expressed in this article are solely those of the authors and do not necessarily represent those of their affiliated organizations, or those of the publisher, the editors and the reviewers. Any product that may be evaluated in this article, or claim that may be made by its manufacturer, is not guaranteed or endorsed by the publisher.
